# Uncovering genetic and non-genetic biomarkers specific for exudative age-related macular degeneration: significant association of twelve variants

**DOI:** 10.18632/oncotarget.23241

**Published:** 2017-12-12

**Authors:** Raffaella Cascella, Claudia Strafella, Giuliana Longo, Michele Ragazzo, Laura Manzo, Cecilia De Felici, Valeria Errichiello, Valerio Caputo, Francesco Viola, Chiara Maria Eandi, Giovanni Staurenghi, Andrea Cusumano, Silvestro Mauriello, Luigi Tonino Marsella, Cinzia Ciccacci, Paola Borgiani, Federica Sangiuolo, Giuseppe Novelli, Federico Ricci, Emiliano Giardina

**Affiliations:** ^1^ Molecular Genetics Laboratory UILDM, Santa Lucia Foundation, Rome, Italy; ^2^ Department of Chemical Pharmaceutical and Biomolecular Technologies, Catholic University Our Lady of Good Counsel, Tirane, Albania; ^3^ Department of Biomedicine and Prevention, Tor Vergata University, Rome, Italy; ^4^ Emotest Laboratory, Pozzuoli, Italy; ^5^ Department of Medical Science, Catholic University Our Lady of Good Counsel, Tirane, Albania; ^6^ UOSD Retinal Pathology PTV Foundation Policlinico Tor Vergata, Rome, Italy; ^7^ U.O. Oculist Foundation IRCCS Cà Granda Maggiore General Hospital, University of Milan, Milan, Italy; ^8^ Department of Clinical Physiopathology, Eye Clinic, University of Turin, Turin, Italy; ^9^ Eye Clinic, Department of Clinical Science Luigi Sacco, Luigi Sacco Hospital, University of Milan, Milan, Italy

**Keywords:** age related macular degeneration, macula, susceptibility, genomic and non-genomic factors

## Abstract

Age-related Macular Degeneration (AMD) represents one of the most sight-threatening diseases in developed countries that substantially impacts the patients’ lifestyle by compromising everyday activities, such as reading and driving. In this context, understanding the prevalence, burden, and population-specific risk/protective factors of AMD is essential for adequate health care planning and provision. Our work aimed to characterize exudative AMD in Italian population and to identify the susceptibility/protective factors (genetic variants, age, sex, smoking and dietary habits) which are specific for the onset of disease. Our study involved a cohort of 1976 subjects, including 976 patients affected with exudative AMD and 1000 control subjects. In particular, the sample cohort has been subjected to a large genotyping analysis of 20 genetic variants which are known to be associated with AMD among European and Asiatic populations. This analysis revealed that 8 genetic variants (*CFH, ARMS2, IL-8, TIMP3, SLC16A8, RAD51B, VEGFA* and *COL8A1*) were significantly associated with AMD susceptibility. Successively, we performed a multivariate analysis, considering both genetic and non-genetic data available for our sample cohort. The multivariate analysis showed that age, smoking, dietary habits and sex, together with the genetic variants, were significantly associated with AMD in our population. Altogether, these data represent a starting point for the set-up of adequate preventive and personalized strategies aimed to decrease the burden of disease and improve the patients’ quality of life.

## INTRODUCTION

AMD affects approximately 21 million people worldwide, and it is the main cause of low vision among people aged 55 years and older [1–2]. In Italy, over 1 million individuals manifest with initial symptoms of the disease, and 63000 new AMD cases are estimated each year [3]. The hallmarks of the disease include the progressive degeneration of the central part of the retina (macula) and drusen formation, changes that ultimately disrupt the cytoarchitectonics of the central retina and cause atrophy or abnormal choroidal neovascularization (CNV). According to the clinical phenotype, AMD is classified as non-exudative or exudative [3–5]. In the early stage of disease, fundus examination reveals the presence of non-exudative lesions such as drusen, areas of Retinal Pigment Epithelium (RPE) depigmentation or pigment clumping. These drusen typically occur with significant pigment changes and accumulation of pigment in the posterior pole. Morphological characteristics of the drusen (size, coalescence, extension) and the occurrence of pigment changes appear to be correlated with the risk of disease evolution. The late stage of AMD is characterized by either the presence of central retinal atrophy, termed geographic atrophy (GA), or the presence of an exudative neovascular lesion, known as choroidal neovascularization (CNV) [5–6]. In the presence of CNV, the abnormal blood vessels typically sprout from the choriocapillaris and can penetrate Bruch's membrane (BrM) into the space beneath the RPE (type 1 CNV) or the subretinal space (type 2 CNV). In some cases, neovascularization appears to initiate from the retinal vessels, induces detachment of the pigment epithelium and finally creates anastomosis with choroidal vasculature (Retinal Angiomatous Proliferation-RAP or type 3 CNV).

Concerning the onset and the evolution of the disease, a number of triggering factors have been described up to date. First, aging is responsible for the loss of approximately 30% of rod photoreceptors and has been directly correlated with disease prevalence [3–4, 7–8]. Second, smoking is known to significantly increase the risk for AMD as a result of the RPE damage caused by the release of oxidative compounds contained in cigarettes [1, 5, 9–10]. The damages caused by cigarettes smoke appeared to be strongly correlated with the exudative form of AMD. Third, dietary habits, especially the consumption of food with antioxidant properties, are usually associated with slower disease progression towards more severe forms [1, 5, 11]. In fact, a number of studies have demonstrated that patients treated with antioxidant nutrients (carotenoids, vitamins, mineral elements and Omega-3 fatty acids) have 25% lower risk of progression towards the advanced stage of AMD and 19% lower risk of vision loss [12]. Moreover, subjects experiencing a healthy lifestyle (meant as regular consumption of antioxidant nutrients, no smoking and physical activity) had 3-fold lower risk of early AMD compared to people having unhealthy lifestyle [13].

Furthermore, familiarity is another susceptibility factor, showing an estimated value of 11% in the presence of affected first-degree relatives [3, 14].

The contribution of genomics to AMD has been so far described through several common and rare variants mapped on different genes. Of these, the following are noteworthy: *CFH* (rs1061170, T/C)*, ARMS2* (rs10490924, G/T)*, IL-8* (rs2227306, C/T)*, C2* (rs547154, C/A; rs9332739, G/C)*, CFB* (rs4151667, T/A)*, C3* (rs2230199, C/G)*, C9* (rs62358361, G/T)*, CFI* (rs4698775, G/T)*, TNFRSF10A* (rs13278062, G/T)*, TIMP3* (rs5749482, C/G), *VEGFA* (rs943080, C/T), *COL8A1* (rs13081855, G/T), *SLC16A8* (rs8135665, G/T), *RAD51B* (rs8017304, A/G), *ADAMTS9* (rs679573, C/T)*, LIPC* (rs920915, C/G)*, APOE* (rs4420638, A/G)*, COL10A1* (rs3812111, A/T)*, IER3-DDR1* (rs1864163, A/G)*, B3GALTL* (rs9542236, C/T)*, TGFBR1* (rs334353, T/G), and *CETP* (rs1864163, G/A) [5, 15–17]. Currently, only *CFH, ARMS2* and *IL-8* are known to be associated with AMD in the Italian population [3, 18–20]. Therefore, we performed a large genotyping analysis to determine the genetic variations associated with Italian patients affected by AMD. Successively, we performed a multivariate analysis, taking into account all genetic data and non-genetic factors (age, sex, smoking and dietary habits). The ultimate goal of the multivariate analysis was to determine the contribution of genetic and environmental factors to the onset of AMD in Italian patients. Moreover, bioinformatic analysis predicted some possible gene interactions at the basis of AMD pathogenesis.

## RESULTS

All of the 1976 samples were genotyped for the selected SNPs. Of the analyzed variants, only 8 SNPs yielded significant association values. As expected, we confirmed that rs1061170 (*CFH*), rs10490924 (*ARMS2*), rs2227306 (*IL-8*) had a strong association with AMD susceptibility in the Italian population. In addition, the biostatistical analysis revealed the association of 5 new SNPs in our population: rs5749482 (*TIMP3*), rs8017304 (*RAD51B*), rs8135665 (*SLC16A8*), rs943080 (*VEGFA*), and rs13081855 (*COL8A1*). The other SNPs tested for statistical association did not yield significant values. The *p* and ORs for the 20 SNPs are reported in Table 1.

**Table 1:** Association analysis results of the 20 selected SNPs

GENES	SNPs	LOCUS	*p*	OR (CI 95%)	EFFECT
*CFH*	rs1061170 (T/C)	1q31	1.05^*^10^−34^	C = 2.3 (2.0–2.6)	risk
*ARMS2*	rs10490924 (G/T)	10q26	2.37^*^10^−42^	T = 2.6 (2.3–3.0)	risk
*IL8*	rs2227306 (C/T)	4q13	1.53^*^10^−05^	T = 1.4 (1.2–1.5)	risk
*TIMP3*	rs5749482 (C/G)	22q12.3	5.96^*^10^−06^	C = 1.6 (1.3–1.9)	risk
*LIPC*	rs920915 (C/G)	15q21	ns	-	-
*APOE*	rs4420638 (A/G)	19p13.2	ns	-	-
*VEGFA*	rs943080 (C/T)	6p21.1	1.13^*^10^−04^	T = 1.3 (1.1–1.4)	risk
*IER3-DDR1*	rs1864163 (A/G)	6p21.3	ns	-	-
*B3GALTL*	rs9542236 (C/T)	13q12.3	ns	-	-
*TGFBR1*	rs334353 (T/G)	9q22	ns	-	-
*ADAMTS9*	rs679573 (C/T)	3p14.1	ns		-
*COL10A1*	rs3812111 (A/T)	6q21	ns	-	-
*CETP*	rs1864163 (G/A)	16q21	ns	-	-
*RAD51B*	rs8017304 (A/G)	14q23	0.007	G = 1.2 (1.0–1.4)	risk
*SLC16A8*	rs8135665 (C/T)	22q13.1	1.89^*^10^−08^	T = 1.6 (1.4–1.9)	risk
*COL8A1*	rs13081855 (G/T)	3q12.1	1.02^*^10^−08^	T = 2.0 (1.5–2.5)	risk
*C2*	rs547154 (C/A) rs9332739 (G/C)	6p21.3	ns	-	-
*CFB*	rs4151667 (T/A)	6p21.3	ns	-	-
*C3*	rs2230199 (C/G)	19p13.3	ns	-	-

The multivariate logistic regression analysis was performed accounting for the 8 associated SNPs, age, sex, smoking and dietary (consumption of fruits and vegetables) habits collected from our Italian subjects.

We performed 3 different levels of multivariate logistic regression analysis to estimate the contribution of genetic and non-genetic factors to AMD susceptibility.

The first level of the multivariate analysis included only the frequency distribution of the 8 associated SNPs. Interestingly, the logistic regression coefficient (R^2^) revealed that genetic factors account for 23% of the disease susceptibility. Moreover, the significant associations of *CFH*, *ARMS2*, *IL-8*, *TIMP3*, *SLC16A8*, *VEGFA* and *COL8A1* were confirmed, while the association of *RAD51B* was not significant. This result suggested that *RAD51B* is a very low risk factor for AMD in the Italian population compared with stronger susceptibility genes.

The second level of the multivariate analysis was performed taking into account only the non-genetic factors, which were age, sex, smoking and dietary habits. The overall contribution of these variables to AMD susceptibility was estimated to be 10% (R^2^). In particular, advanced age and smoking status were strongly associated with a higher risk of AMD, while the regular consumption of fruits and vegetables showed to be protective from the disease. The association between sex and disease susceptibility was non-significant at this level of analysis. The *p* and ORs for each non-genetic variable are reported in Table 2.

**Table 2:** Multivariate analysis performed on the non-genetic variables

NON-GENETIC VARIABLES	*p*	OR (CI 95%)	EFFECT
Age	1.58^*^10^−36^	1.4 (1.3–1.5)	risk
Smoking habit	1.31^*^10^−4^	1.4 (1.2–1.7)	risk
Dietary habits (fruits&vegetables)	1.43^*^10^−4^	0.6 (0.5–0.8)	protective
Sex	ns	-	-

The third level of the multivariate analysis included all genetic and non-genetic factors taken into consideration in our work. The logistic regression coefficient (R^2^) suggested that all of the tested variables were able to describe up to 29% of disease susceptibility. Overall, *CFH* and *ARMS2* remained the most significant risk factors for AMD, while *RAD51B* was identified as the least relevant gene. Surprisingly, at this step of the analysis, sex appeared to be slightly associated with the disease. In particular, females appeared to be at lower risk for AMD with respect to males. The *p* and ORs for the genetic and non-genetic variables are reported in Table 3.

**Table 3:** Multivariate analysis performed on genetic and non-genetic variables

CONSIDERED VARIABLES	*p*	OR (CI 95%)	EFFECT
***CFH* (rs1061170, T/C)**	1.29^*^10^−23^	C = 2.7 (2.2–3.3)	risk
***ARMS2* (rs10490924, G/T)**	2.19^*^10^−26^	T = 3.1(2.5–3.9)	risk
***IL8* (rs2227306, C/T)**	2^*^10^−4^	T = 1.4 (1.1–1.7)	risk
***TIMP3* (rs5749482, C/G)**	4^*^10^−4^	C = 1.7 (1.5–2.2)	risk
***VEGFA* (rs943080, C/T)**	0.032	T = 1.2 (1.0–1.5)	risk
***RAD51B* (rs8017304, A/G)**	ns	-	-
***SLC16A8* (rs8135665, C/T)**	5.71^*^10^−11^	T = 2.1 (1.7–2.7)	risk
***COL8A1* (rs13081855, G/T)**	0.014	T = 1.5 (1.1–2.1)	risk
Age	2.48^*^10^−19^	1.4 (1.3–1.5)	risk
Smoking habit	7.85^*^10^−5^	1.7 (1.3–2.2)	risk
Dietary habits (fruits&vegetables)	0.015	0.6 (0.4–0.9)	protective
Female sex	0.04	0.7 (0.5–0.9)	protective

**Figure 1:**
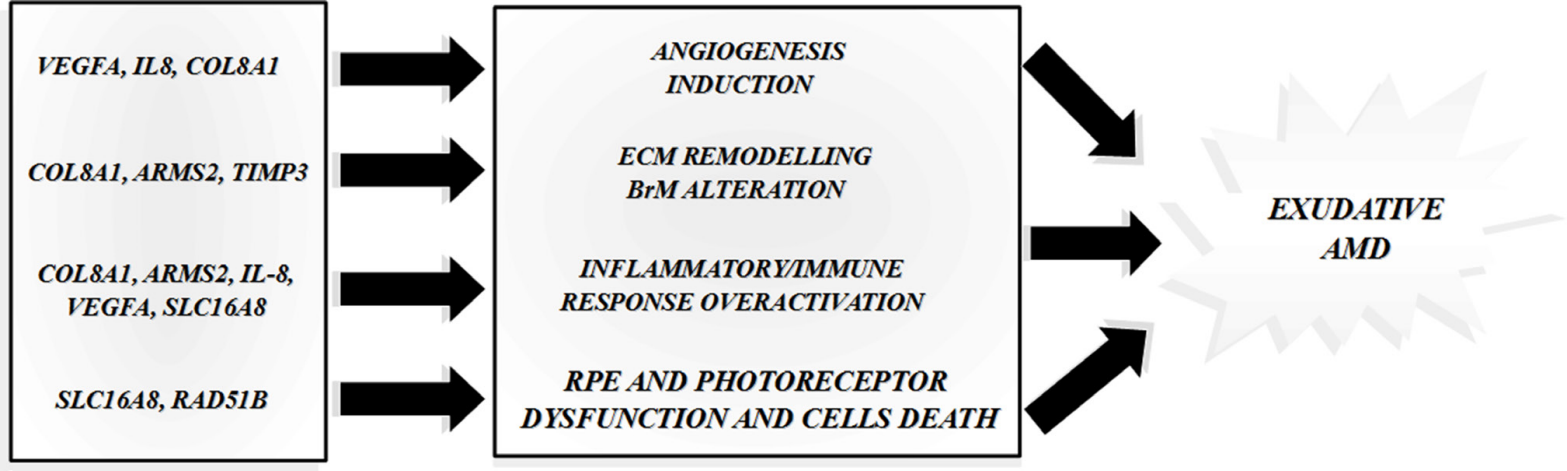
Predicted interactions among the AMD-associated genes The figure shows the main gene interactions derived by the prediction analysis with the bioinformatic tools.

The bioinformatic analysis of the gene interactions through WEB-based GEne SeT AnaLysis Toolkit and DAVID Bioinformatic Database, provided different results depending on the selected enrichment tool (GO Analysis, Pathway Commons Analysis and Transcription Factor Target Analysis). With regard to biological processes, the GO Analysis revealed significant interactions for all the genes of interest (Figure 1). The Pathway Commons Analysis showed that *VEGFA* and *IL-8* share different pathways (*p* ranging from 0.02 and 0.008), especially those involved in signaling, metabolism and regulation of immune and inflammatory events. In addition, *RAD51B* and *SLC16A8* showed a significant interaction (*p* = 0.002) in the modulation of the haemostasis/coagulation pathway. Transcription Factor Target Analysis reported significant associations with some transcription factors (TF). Of these TFs, the most relevant target was NFAT, which was associated with *VEGFA* and *COL8A1* (*p* = 0.004).

With regard to gene/phenotype interactions, analyses performed using SPSS software demonstrated an association between *VEGFA* and type 1 CNV, with *p* = 0.006 and OR = 1.97 (95% CI: 1.20–3.23); but no significant associations were observed for the type 2 CNV subtype. However, the other genes did not appear to affect a specific subtype of exudative AMD, rather they should be regarded as susceptibility genes regardless of the disease phenotype.

## DISCUSSION

The contribution of genomics to the risk of developing AMD has been extensively explored among global populations, particularly in European and Asian groups. According to recent data and disease prevalence estimates, up to 34 loci are known to account for approximately 46–71% of the risk of AMD globally [1, 17].

This study was designed to assess the specific contribution of genetic and environmental factors to the onset of exudative AMD in Italian population. To this end, 1976 Italian subjects were genotyped for 20 variants, which have previously been associated with the disease in European and Asiatic populations. Given our results, only 8 genetic variants (*CFH*, *ARMS2*, *IL-8*, *TIMP3*, *SLC16A8*, *RAD51B*, *VEGFA* and *COL8A1*) out of 20 reached the significance threshold fixed at *p* < 0.05. As expected, *CFH* and *ARMS2* appeared to be the most associated with AMD. The association between *IL-8* and the disease was confirmed in our cohort, although it has never been tested in other populations.

**Figure 2:**
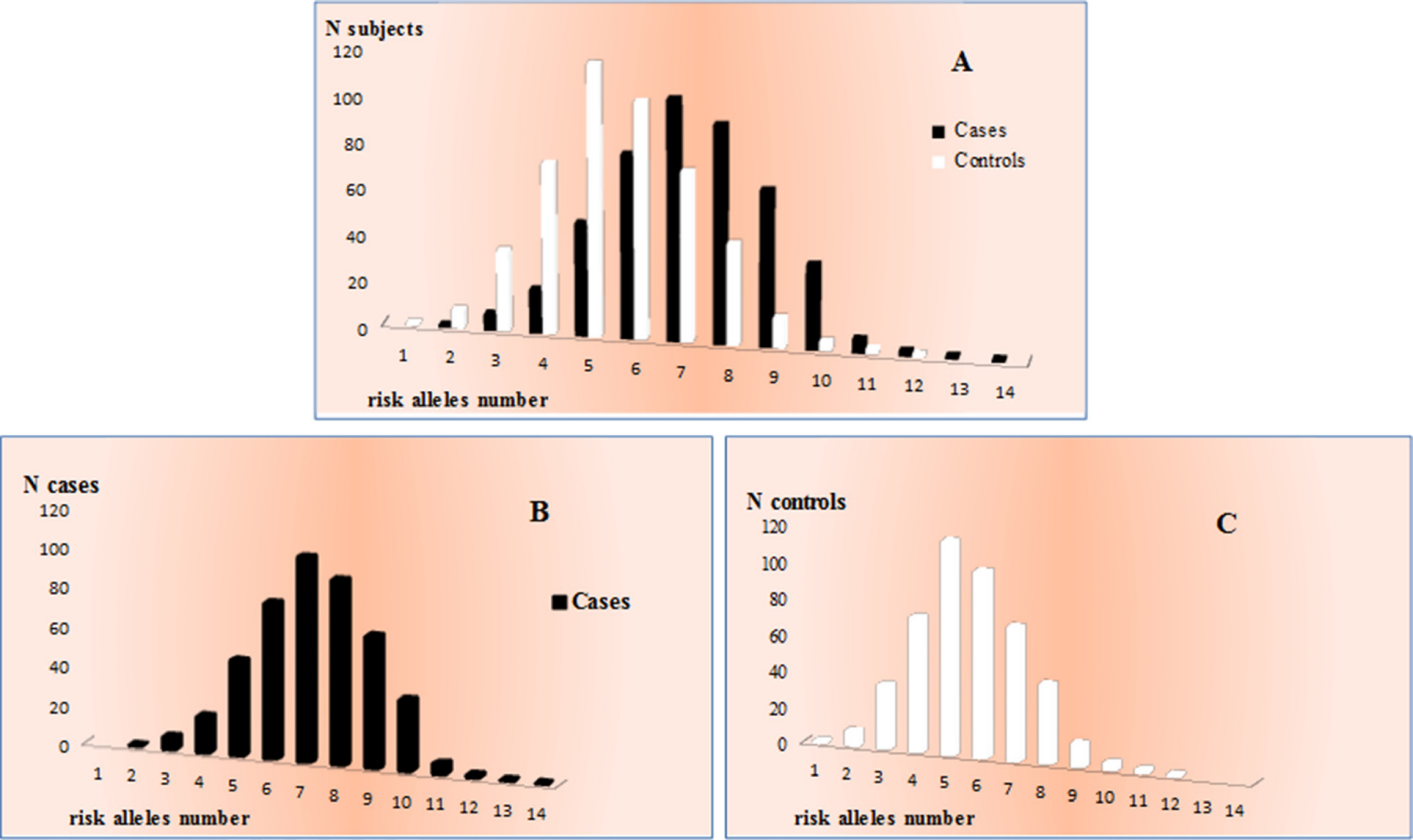
Distribution of the number of risk alleles between cases and controls (**A**) allele frequency distribution in the two groups. (**B**) allele frequency distribution in cases. (**C**) allele frequency distribution in control subjects.

According to multivariate logistic regression analysis, the identified genetic factors were estimated to account for 23% of AMD susceptibility in Italian cohort. It is important to note that this estimate accounted for all of the associated genes except for *RAD51B*. Thus, *RAD51B* can be considered a very low risk factor for AMD, and it actually loses its significance when taken together with more strongly associated genes (*CFH*, *ARMS2*, *IL-8*, *TIMP3*, *SLC16A8, VEGFA* and *COL8A1*). Moreover, the multivariate logistic regression analysis reported that *CFH* and *ARMS2* accounted for 20% of the overall genetic susceptibility to AMD, while the remaining 3% was attributable to *IL-8*, *TIMP3*, *SLC16A8, VEGFA* and *COL8A1*. Next, we evaluated the distribution of the number of risk alleles between cases and controls. As expected, the frequency distribution in the two groups showed different trends (Figure 2A), with a higher number of risk alleles in cases (Figure 2B) with respect to control subjects (Figure 2C). In fact, considering 7 as the median point out of 14 possible variant combinations, we observed a higher number of cases with at least 7 risk alleles compared with the control group. Thus, the presence of an increased number of risk alleles in AMD patients allowed us to presume a cumulative effect of genetic variants in determining the disease phenotype.

The secondary goal of the study was to estimate the contribution of non-genetic factors to the risk of AMD. To this end, we decided to consider environmental variables (age, sex, smoking and dietary habits) that are known to strongly impact the onset/progression of disease. As expected, advanced age and smoking status were found to be strongly associated risk factors for AMD, while regular consumption of fruits and vegetables represented a helpful source for preventing disease onset/progression. However, our multivariate analysis showed that non-genetic factors accounted for 10% of disease, in contrast with the 19–37% described in the literature [1].

Afterwards, genetic and non-genetic factors were combined in one multivariate analysis, in order to provide a comprehensive overview of AMD in Italian population. Our results suggested that all tested variants were able to cover 29% of disease. In particular, genes (*CFH*, *ARMS2*, *IL-8*, *TIMP3*, *SLC16A8, VEGFA* and *COL8A1*), old age and smoking status contribute to susceptibility, while female sex and regular consumption of fruits and vegetables protect from AMD development.

Interestingly, our results highlighted a prominent difference between the Italian and global population, in which the genetic contribution has been estimated to range from 46% to 71% [1]. These data suggest that Italian-associated genes may have a greater impact on disease in our population. In fact, these genes are sufficient to account for 23% of susceptibility, in contrast to the 34 loci required to account for 46–71% of AMD risk across the global populations. Overall, our results suggest that AMD onset in the Italian population may be regulated by distinct interaction patterns involving few genes. To this end, we explored potential interactions of the associated genes and their function. In particular, *CFH* functions as an essential inhibitor of complement activation, and the homozygous genotype for the rs1061170 risk allele has been shown to increase the activation of the complement cascade [4–5, 21–22]. Although the role of *ARMS2* needs to be clarified, studies performed on animal models have reported that it may be involved in angiogenesis, extracellular matrix mineralization, and transforming growth factor-beta signaling [4, 23–24].

In addition, ARMS2 may interact with pro-inflammatory molecules, such as IL-6, IL-8, TNF-α, C3 and C5 [23]. IL-8 is known to be a primary mediator of angiogenesis, and it is fundamentally involved in the chemotaxis of immune cells (neutrophils and lymphocytes) to the site of inflammation [20]. Regarding the activation of angiogenesis, VEGFA is recognized as one of the most important growth factors triggering this mechanism [1, 5]. Therefore, it is not surprising that our bioinformatic analysis suggested significant possible interactions between *IL-8*, *VEGFA*, *CFH* and *ARMS2*. Thus, the involvement of *CFH*, *ARMS2*, *IL-8* and *VEGFA* in the inflammatory, angiogenic and tissue homoeostasis pathways fits perfectly with the clinical phenotype displayed by patients affected with exudative AMD. In addition, the gene/phenotype interaction analysis revealed that the risk allele (T) of *VEGFA* (rs943080) is more frequent among patients with type 1 CNV compared with those with the type 2 CNV subtype. Importantly, rs943080 maps to a highly evolutionary conserved region of chromosome 6. In particular, the alternative allele C is believed to disrupt the binding site of cone-rod homeobox (CRX), which is an essential transcription factor that is highly expressed in RPE and retinal ganglion cells [25–26]. Thus, the disruption of the CRX binding site may reduce the expression of *VEGFA* beneath the RPE monolayer and decrease neovascularization in type 1 exudative AMD. In study involving 223 patients with neovascular AMD, subjects carrying the *VEGFA* rs943080 risk genotype (TT) had 1.8-fold higher expression of *VEGFA* compared with patients with the protective *VEGFA* rs943080 genotype (CC) variant (*p* = 0.012) [26]. Based on these data, *VEGFA* genotyping may help clinicians to recognize some clinical features indicative of type 1 exudative AMD. Moreover, the TT genotype of *VEGFA* rs943080 has been associated with a poorer response to anti-VEGFA therapy compared with the low-risk genotype (CC) [26–27]. These data indicate *VEGFA* may be a pharmacogenetic biomarker that influences the degree of response to anti-VEGFA treatments; the biomarker may be useful to determine the dosage and administration frequency according to the patient's genotype.

Interestingly, the bioinformatic analysis showed a significant interaction between *VEGFA* and *COL8A1*. The homonymous protein encoded by *COL8A1* is a major component of Descemet's membrane of corneal and blood vessel endothelial cells. COL8A1 permits the migration and proliferation of vascular smooth muscle cells, is involved in the maintenance of vessel wall integrity and structure and participates in extracellular matrix (ECM) remodeling and angiogenesis [4–5, 17]. We therefore believe that genetic variants in *COL8A1* may result in altered protein, causing more fragile vessels, vessel disruption and the onset of edema that is normally associated with wet AMD. Another possible role of *COL8A1* in AMD pathogenesis may lie in its participation in ECM remodeling pathways, which thereby trigger the progression of angiogenesis.

Moreover, the TF target bioinformatic analysis revealed a significant relationship between *COL8A1* and *VEGFA* with NFAT transcription factors. The NFAT TFs are a family of proteins mainly involved in immune responses and VEGF-induced angiogenesis and therefore have a potential role in the aetiopathogenesis of AMD [28].

Regarding interactions among *TIMP3*, *VEGFA, RAD51B* and *SLC16A8,* bioinformatic data indicated significant interactions only for *RAD51B, VEGFA* and *SLC16A8*. In particular, *TIMP3* is an inhibitor of metalloproteinase 3, which is involved in the degradation of extracellular matrix. It is present in BrM, where it is essential for ECM maintenance and remodeling [4, 6, 17]. In this context, AMD-associated variations in ECM proteins can alter the structure and permeability of the BrM, making it more susceptible to aging and environmental abuse. *RAD51B* is essential for the activation of DNA-repair mechanisms and may play a role in the induction of RPE and the photoreceptor cell death at the root of AMD pathogenesis [5]. *SLC16A8* encodes a transporter protein comprising many short-chain monocarboxylates that spans the membrane. This protein is specifically expressed by the RPE and a deficit in lactate transport may cause the retinal acidification and photoreceptor dysfunction [17]. Altogether, our bioinformatic results provided interesting insights into the interactions among AMD-associated genes, which may thereby trigger alterations of BrM structure and permeability, the induction of angiogenesis and the damage caused by aging and environmental factors. Clearly, further molecular and cellular experiments are necessary to verify the effective relationships between *CFH*, *ARMS2*, *IL-8*, *TIMP3*, *SLC16A8, VEGFA* and *COL8A1* in the development and progression of the disease.

Our work provides a general overview of the genetic and non-genetic factors (which contribute to the onset of exudative AMD in Italian population. The extensive analysis performed on 1976 subjects highlighted 8 genetic variants and 4 non-genetic factors associated with exudative AMD.

Regarding genetic susceptibility, our findings differed from global studies that have described many more associated variants than the 8 reported above. In addition, the bioinformatic analysis revealed interesting insights into the biological functions and interactions among the genes associated with AMD in our cohort. Our findings suggest that *CFH*, *ARMS2*, *IL-8*, *TIMP3*, *SLC16A8, RAD51B*, *VEGFA* and *COL8A1* may contribute to the onset of wet AMD at different levels, particularly the following: induction of angiogenesis; alteration of ECM remodeling mechanisms and of BrM integrity and permeability; modification of RPE and photoreceptor cell activities; and over-activation of inflammatory and immune responses.

**Figure 3:**
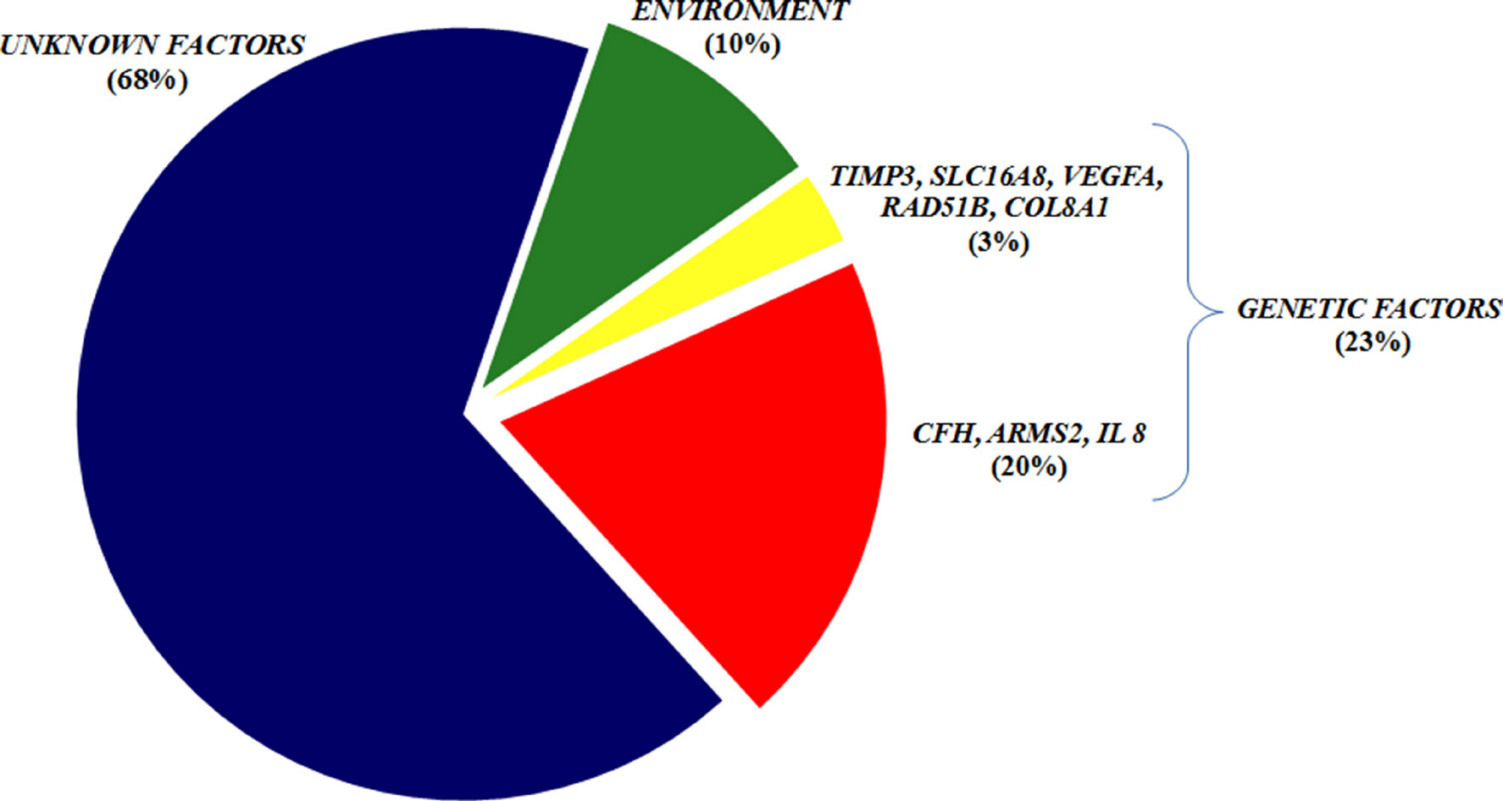
The AMD susceptibility within the Italian population The chart illustrates the main components able to describe the genetic and non-genetic variables contributing to the disease susceptibility in our population.

Regarding environmental effects on the disease, our data confirmed previously reported findings. As expected, smoking status and advanced age were two of the major risk factors for AMD, while regular consumption of fruits and vegetables was as preventative factor. On the other hand, the association between sex and AMD was discordant with previous data. In our study, women had a lower risk of disease, while other studies have reported non-significant or conflicting data on this subject. However, the association value was very weak in our study, and may not be relevant in comparison with other environmental factors. Overall, our study showed that AMD susceptibility within the Italian population is contributed to by genetic variants, accounting for 23% of disease and non-genetic variants, accounting for 10% of AMD (Figure 3). The remaining 67% of disease susceptibility is still a matter of investigation. Other promising biomarkers certainly contribute to disease, among which epigenetic factors are of great interest. In this context, special attention should be paid to methylation patterns and miRNAs, since they are likely to be involved in the mechanisms underlying the onset and progression of disease. In particular, methylation seems to impact the proinflammatory activity and oxidative response which are significantly increased in AMD pathogenesis [1, 29]. MiRNAs have been closely connected with angiogenic, inflammatory and cell survival mechanisms which are usually disrupted in case of disease [5, 29]. Although the relationship with AMD needs to be clarified, epigenetic modifications represent the most promising biomarkers for the prediction of the risk/ protection to exudative AMD as well as for the development of new drugs.

## MATERIALS AND METHODS

### Study cohort and DNA extraction

This study enrolled 1976 individuals, including 976 wet-AMD cases and 1000 control subjects. Cases were recruited from the Ophthalmology Unit of the PTV General Hospital of Rome, U.O. Oculist Foundation IRCCS “Cà Granda” Maggiore General Hospital of Milan, Department of Clinical Physiopathology of the University of Turin, Department of Clinical Science of Sacco Hospital, of Milan. Patients were selected following specific inclusion criteria: male or female subjects over the age of 50 years, diagnosis of exudative AMD, clear media in order to guarantee good quality images. The exclusion criteria included: signs of any other macular condition predisposing to CNV, any previous intraocular treatment, including laser photocoagulation and photodynamic therapy with verteporfin (PDT), cataract surgery, allergy to fluorescein. The control subjects were recruited from the UOSD SIMT and the Ophthalmology Unit of the PTV General Hospital of Rome. Recruited subjects were asked to reply to a questionnaire in order to classify their smoking and dietary habits. Subjects who have never smoked or quit smoking ≥ 5 years before recruitment were classified as non-smokers, otherwise they were considered as smokers. Dietary habits were sorted into regular intake of fruit and vegetables (≥ 3 times per week) and unusual assumption (≤ 2 times per week). All of the data related to the characterization of the study cohort are summarized in Table 4.

**Table 4:** Collection of data concerning the subjects enrolled in the study

DATA	CASES	CONTROLS
Age	± 77 years old	±72 years old
Sex	F: 54%	F: 56%
	M: 46%	M: 44%
Type of CNV	Type 1:53%	-
	Type 2: 47%	
Dietary habits	R: 86%	R: 79%
	S: 14%	S: 21%
Smoking habit	Y: 47%	Y: 47%
	N: 53%	N: 53%

Concerning smoking habits, non-smokers status was assigned to those subjects who never smoked or quit smoking ≥ 5 years before recruitment, otherwise they were considered as smokers. Dietary habits were sorted according to the frequency of fruits and vegetables intake (≥ 3 times/week for regular consumption and ≤ 2 times/week for unusual consumption). Legend: F= female; M: male; R: regular; S: seldom; Y: yes; N: not.

The study was approved by the ethics committee of the University of Rome “Tor Vergata” and was performed according to the Declaration of Helsinki. All participants provided signed informed consent. Blood samples were obtained from all subjects in order to extract genomic DNA. Genomic DNA extraction was performed using the EZ1 Advanced XL automated extractor and the EZ1 DNA Blood 200 μl Kit (Qiagen).

### Genotyping analysis

All of the samples (1976 individuals) were genotyped for the 22 selected SNPs, which were as follows: *CFH* (rs1061170, T/C)*, ARMS2* (rs10490924, G/T)*, IL-8* (rs2227306, C/T)*, C2* (rs547154, C/A; rs9332739, G/C)*, CFB* (rs4151667, T/A)*, C3* (rs2230199, C/G)*, CFI* (rs4698775, G/T)*, TNFRSF10A* (rs13278062, G/T)*, TIMP3* (rs5749482, C/G), *VEGFA* (rs943080, C/T), *COL8A1* (rs13081855, G/T), *SLC16A8* (rs8135665, G/T), *RAD51B* (rs8017304, A/G), *ADAM* (rs679573, C/T)*, LIPC* (rs920915, C/G)*, APOE* (rs4420638, A/G)*, COL10A1* (rs3812111, A/T)*, IER3-DDR1* (rs1864163, A/G)*, B3GALTL* (rs9542236, C/T)*, TGFBR1* (rs334353, T/G), and *CETP* (rs1864163, G/A). The *CFI* and *TNFRSF10A* genotype analyses were limited by technical issues, and therefore the required molecular experiments were not successfully completed.

All samples were genotyped using the TaqMan assay on a 7500 Fast Real Time PCR device according to the manufacturer's instructions (Applied Biosystems). The genotyping results were interpreted using Sequence Detection System 2.1 software (Applied Biosystems). Each Real Time PCR run was performed using a negative control and three positive control samples previously confirmed by direct sequencing (BigDye Terminator v3.1, BigDyeXTerminator) on ABI3130xl (Applied Biosystems).

### Biostatistical and bioinformatic analysis

The genotyping results were subjected to biostatistical analysis to evaluate associations with AMD. First, the genotyping data reported in our cohort were tested to confirm the Hardy-Weinberg equilibrium (*p* > 0.05). Afterwards, the association of the genotyped SNPs were measured by calculating the *p*-value (*p*) through 2×2 contingency tables (http://www.physics.csbsju.edu/stats/contingency_NROW_NCOLUMN_form.html). The statistical associations were considered significant when *p* < 0.05 based on the 95% confidence interval. The strength of the associations was determined by calculating the Odd Ratio (OR, http://www.hutchon.net/ConfidOR.htm).

The overall contribution of each genetic and non-genetic factor to AMD susceptibility was evaluated through a multivariate logistic regression analysis (stepwise method). The presence/absence of AMD was considered the dependent variable. First, the contributions of genetic factors associated with AMD in our cohort were determined. To this end, the analysis included *CFH*, *ARMS2*, *IL-8*, *TIMP3*, *COL8A1*, *SLC16A8*, *VEGFA*, and *RAD51B* as independent variables. Second, the contribution of non-genetic factors was estimated, taking into account age, sex, smoking and dietary habits as independent variables. The last analysis was performed considering both genetic and non-genetic factors as independent variables. At each level of the multivariate logistic regression analysis, the R^2^ (logistic regression coefficient) indicated the contribution of the considered variables to AMD susceptibility. The cut-off for statistical significance was set at a *p* < 0.05. The multivariate analysis was performed using the SPSS program, ver. 19 (IBM Corp, Armonk, NY, USA).

### Bioinformatic evaluation of genes interactions

All the associated genes were subjected to a bioinformatic analysis aimed to evaluate the potential presence of specific interactions among them. To this end, the WEB-based GEne SeT AnaLysis Toolkit [30] and DAVID Bioinformatic Database version 6.8 Beta [31–32] were utilized. The software incorporates information from different public resources and provides information about the possible biological and molecular pathways involving the genes of interest. The analysis was performed according to the following enrichment tools: GO Analysis, Pathway Commons Analysis and Transcription Factor Target Analysis. The results were considered significant with a cut-off of *p* < 0.05.

## Abbreviations

AMD: Age-related Macular Degeneration
SNPs: Single Nucleotide Polymorphisms
CNV: Choroidal NeoVascularization
RPE: Retinal Pigment Epithelium
GA: Geographic Atrophy
BrM: Bruch's membrane
TF: Transcription Factors
CRX: Cone-Rod homeoboX
*p*: *p*-value
OR: Odd Ratio
